# Complete Genome Sequences of Cluster S Mycobacteriophages Beelzebub, Raela, and RedRaider77

**DOI:** 10.1128/mra.01173-22

**Published:** 2022-12-12

**Authors:** Kristina Sevcik, Peace Preston, Michaela Aulner, Byron Noordewier, Sara S. Tolsma

**Affiliations:** a University of Iowa Carver College of Medicine, Iowa City, Iowa, USA; b A. T. Still University, Kirksville College of Osteopathic Medicine, Kirksville, Missouri, USA; c Department of Biology, Northwestern College, Orange City, Iowa, USA; Queens College Department of Biology

## Abstract

We isolated three mycobacteriophages that belong to cluster S, namely, Beelzebub, Raela, and RedRaider77. Annotation revealed a genome structure typical of cluster S phages, including an atypical location of two minor tail protein genes in the right arm of these viral genomes.

## ANNOUNCEMENT

Bacteriophages are abundant infectious particles that have been found in nearly every ecosystem investigated (1). They represent a population that is rapidly evolving (2, 3). Here, we report the genomic structures of three members of this dynamic population, contributing to the growing understanding of phage evolution and interactions between phages and their bacterial hosts (4).

**FIG 1:**
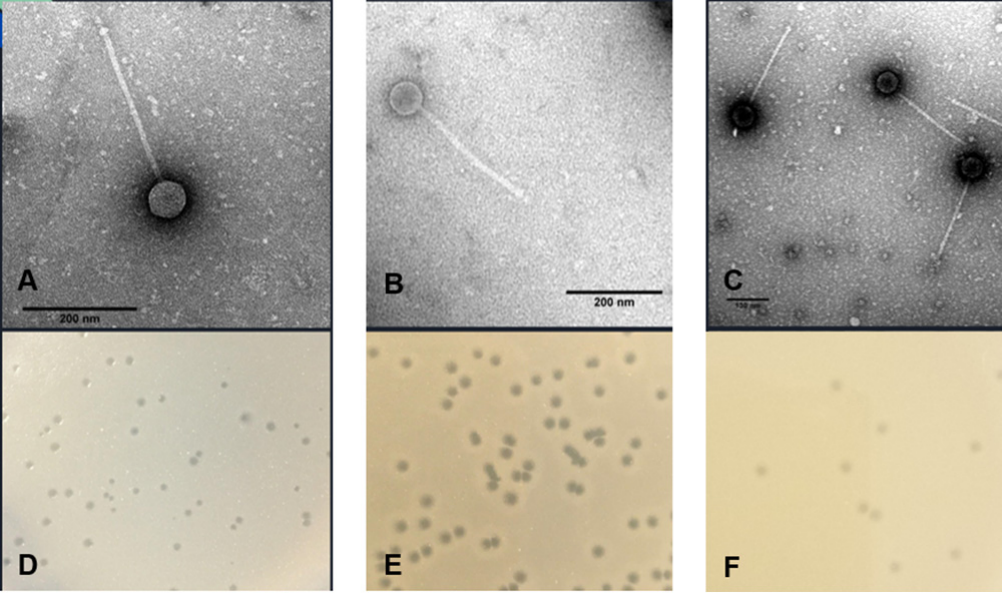
(A to C) Transmission electron micrographs of phages stained with 1% uranyl acetate, i.e., Beelzebub (head, 54-nm diameter; tail, 335 nm [*n* = 1]) (A), Raela (head, 71-nm diameter; tail, 284 nm [*n* = 1]) (B), and RedRaider77 (head, 86-nm diameter; tail, 300 nm [*n* = 2]) (C), analyzed using ImageJ (21). Magnification, ×25,000 to ×30,000. (D to F) Plaque characteristics of phages Beelzebub (plaque diameter range, 0.6 to 1.5 mm [mean diameter, 1.2 mm] [*n* = 10]) (D), Raela (plaque diameter range, 1.6 to 2.4 mm [mean diameter, 2.2 mm] [*n* = 10]) (E), and RedRaider77 (plaque diameter range, 1.3 to 2.1 mm [mean diameter, 1.6 mm] [*n* = 10]) (F) after 24 h at 37°C.

We discovered three mycobacteriophages, each of which was isolated from 15 g of surface soil using standard methods (Table 1) (5). Briefly, soil samples were washed with 7H9 liquid medium, and phages in the filtered (0.2-μm pore size) wash were isolated and purified with at least three rounds of plating in 7H10 top agar overlays with Mycobacterium smegmatis mc^2^155, producing clear plaques after 24 h at 37°C (Fig. 1). Examination with negative-staining electron microscopy revealed that these phages have siphovirus morphology, with icosahedral heads and flexible, noncontractile tails (Fig. 1).

**TABLE 1:** Isolation details, sequencing details, and genomic characteristics of phages Beelzebub, Raela, and RedRaider77

Phage name	Soil sample collection site coordinates	Approximate shotgun coverage (×)	Total no. of sequencing reads	Genome size (bp)	Genome end	G+C content (%)	No. of protein-coding genes	Cluster assignment
Beelzebub	40.76289N, 96.62133W	636	298,002	65,529	3′ single-stranded overhang (5′-GCGCGCAGCGC-3′)	63.4	118	S
Raela	43.35896N, 98.41135W	3,997	1,860,932	65,380	3′ single-stranded overhang (5′-GCGCGCAGCGC-3′)	63.4	117	S
RedRaider77	38.885732N, 104.707011W	249	225,154	64,827	3′ single-stranded overhang (5′-GCGCGCAGCGC-3′)	63.4	115	S

Phage DNA was isolated from high-titer lysates with the Wizard DNA clean-up system (Promega) and then concentrated with the DNA Clean and Concentrator kit (ZYMO Research). Sequencing libraries were prepared from genomic DNA with a NEBNext Ultra II FS kit with dual-indexed barcoding and sequenced on an Illumina MiSeq system; sequencing produced 150-base, single-end reads, which were assembled using Newbler v2.9 with default settings (6) and in each case yielded a single phage contig. The contigs were checked for completeness, accuracy, and phage termini using Consed v29.0 with default settings (7). Genome characteristics and sequencing details are reported in Table 1.

Annotation was performed using Glimmer v3.02, GeneMark v2.5, Phamerator, Starterator (http://phages.wustl.edu/starterator), HHpred, DNA Master v5.23.2 (http://cobamide2.bio.pitt.edu), and BLASTp (8–12). No tRNA genes were identified when the genomes were screened using ARAGORN and tRNAscan-SE (13, 14).

The phages were assigned to cluster S based on gene content similarity (GCS) values of ≥35% with respect to sequenced bacteriophages present in the Actinobacteriophage Database (phagesDB) (15, 16). We identified 118 putative protein-coding genes in Beelzebub, 117 in Raela, and 115 in RedRaider77 (Table 1).

The three genomes show considerable similarity with the exception of a 2-kb region between genes 7 and 14, which showed significant divergence in sequences and annotated genes. The genomes of both Beelzebub and Raela encode a putative HNH endonuclease (genes 55 and 51, respectively) that is absent in RedRaider77, although all three genomes encode a putative HNH endonuclease downstream (genes 72, 68, and 68 in Beelzebub, Raela, and RedRaider77, respectively) (17). The three phage genomes encode putative proteins proposed to play a role in the arms race between phages and their bacterial hosts, including *O*-methyltransferase (genes 89, 86, and 84 in Beelzebub, Raela, and RedRaider77, respectively) and methyltransferase (absent in Beelzebub, gene 87 in Raela, and 85 in RedRaider77) (18). The genomes also include multiple putative glycosyltransferases (genes 90, 91, and 93 in Beelzebub, genes 88 and 90 in Raela, and genes 86 and 88 in RedRaider77) (19).

As first observed in phage Marvin (also in cluster S), these genomes have two predicted minor tail protein genes (genes 53 and 54 in Beelzebub and genes 49 and 50 in both Raela and RedRaider77) in a noncanonical location among nonstructural genes in the right arm of the genome (20).

### Data availability.

The complete genome sequences of phages Beelzebub, Raela, and RedRaider77 are available in GenBank under accession numbers MK061407, MK279842, and MK305892, respectively. The raw sequencing reads are available in the NCBI SRA under accession numbers SRX17910050, SRX17910052, and SRX17910053.
